# Supplementary material for: Are carbon emissions trading and green financial instruments synergistic? -Comprehensive quantitative research based on content analysis
Source: PLoS One. 2024 Mar 7;19(3):e0298601. doi: 10.1371/journal.pone.0298601 (PMC10919601; doi:10.1371/journal.pone.0298601)
Supplement: S1 Data — (ZIP) [file pone.0298601.s001.zip › Data/地级市Z_F工作报告.zip!/╦─┤¿/╡┬╤⌠╩╨/└┤╫╘╣1⁄2╓┌║┼í╛┬φ┐╦╩2╛▌═°í┐_2019.docx]

各位代表：

现在，我代表市人民政府，向大会报告工作，请予审议，并请市政协委员和列席人员提出意见。

2018年工作回顾

过去的一年，我们深入贯彻习近平新时代中国特色社会主义思想和习近平总书记对四川工作系列重要指示精神，全面贯彻党的十九大精神，认真落实省委十一届三次全会和市委八届七次全会决策部署，扎实开展“大学习、大讨论、大调研”活动，在中共德阳市委的坚强领导下，在市人大、市政协的监督支持下，与全市人民一道，创新实干，开拓奋进，顺利完成市八届人大第三次会议确定的主要目标任务，为建设全省经济副中心城市打下了良好基础。

经济发展迈上新台阶。地区生产总值跨入“2000亿俱乐部”，达到2213.9亿元、增长9%。全社会固定资产投资同口径增长12.4%，一般公共预算收入同口径增长10.1%，规上工业增加值增长10.8%，服务业增加值增长10%，社会消费品零售总额增长12.5%，进出口总额增长21.9%、增速提高32.4个百分点，城乡居民人均可支配收入分别增长8.2%、9%。项目建设成效显著，237个省市重点项目完成投资514亿元。招商引资实现历史性突破，引进京东西南智能运营结算中心、深兰科技西南总部2个百亿项目，新签约项目投资额达1159亿元、居全省第二。罗江在全省县级经济排位中上升9位。高规格召开民营经济健康发展大会，出台促进民营经济健康发展26条意见，减税47亿元、降费1.8亿元，民营经济增加值增长9.2%，7家民营企业入围全省百强。

高质量发展迈出新步伐。产业结构持续优化，三次产业结构为11.0:48.4:40.6。工业转型步伐加快。规上工业增加值增速创四年新高，利润总额增长20.3%。战略性新兴产业利润总额增长25.8%。完成工业投资445.4亿元、增长9.6%。规上企业单位增加值能耗下降9%左右，提前三年完成省定“十三五”目标。入围省大企业大集团百强企业3户，国机重装挂牌成立。获批筹建国家发电设备机械零部件产品质量监督检验中心（四川）。现代服务业发展加快。旅游业增长35%，德阳文旅产品荣获“2018中国特色旅游商品大赛”三项金奖，中江挂面荣获“2018中华品牌商标博览会”金奖。电子商务实现交易额532亿元、居全省第四。金融机构本外币贷款余额1459.4亿元、增长11.4%，新引进商业银行3家，挂牌开业农商银行3家，天府（四川）股权交易中心挂牌企业数居全省第二。农业现代化水平稳步提升。综合机械化水平达73%，良种覆盖率达96%，水稻单产创历史新高，粮食总产量194.9万吨。成立乡村振兴农民大学，培育新型职业农民2500余人。获批国家农业科技园区1个、中国美丽休闲乡村1个、国家级农业产业化龙头企业1家、国家级农民专业合作社示范社9家。“蜀道”获全省优质品牌。建成亚洲最大雪茄烟原料生产基地。创新能力持续增强。高新技术产业实现主营业务收入1050亿元，R&D占GDP比重达2.69%。“华龙一号”核电机组成功研制，获国家首台（套）重大技术装备保险补偿2.98亿元、占全省93%。获中国专利优秀奖5项、省科技进步奖13项。新增中国驰名商标1件。新增省级重点实验室1个、企业技术中心13个、科技成果转移转化示范区2个。德阳经开区获批国家高端装备制造业标准化试点，绵竹经开区获批省级高新区。什邡获批国家首批创新型县，旌阳、中江被确定为国家知识产权强县工程试点县。新增创新创业载体12家，入孵企业850家。

区域发展取得新进展。成德同城化取得阶段性重大成效。签订成德眉资同城化发展合作协议，启动编制“成德同城化空间发展规划”，市域铁路规划纳入“成都平原城市群轨道交通规划”。成德动车实现公交化营运。天府大道北延线开工建设，成都经济区环线德阳段加快建设，中金快速通道全线通车。成都农交所德阳所实现交易额11.7亿元。成德工业园实现工业产值110亿元、增长50.7%。成德共建铁路物流港、军民融合航空产业园、三星堆与金沙联合申遗、自贸区协同改革先行区创建等工作有序推进。实现医保直接结算、户籍网上迁移一站式办结。德绵一体化正式启动，签署一体化发展合作协议。城乡发展提质加速。全市常住人口城镇化率达52.35%%，中心城区入选省级“城市双修”试点市。实施城建项目160个，开工建设柳梢堰湿地公园，拆除违法建筑3.8万平方米，新（改）建公厕155座。主城区到罗江区公交开通运营。基础设施建设加快，实施重点交通项目21个，德罗、德中等项目开工建设，新（改）建农村公路410公里，八角水库枢纽工程竣工蓄水，华强沟水库、石泉水库加快建设。“百镇建设行动”有序推进，创建省级特色小城镇1个。

改革开放增添新动能。全面深化改革稳步推进，完成国家级改革试点11项、省级6项，改革规划任务完成率达94.6%，4个案例入选《四川改革试点典型案例集》，罗江农联改革、“四位一体”投资建设新模式在全省推广。全面创新改革试验任务基本完成，7条经验在全国全省推广，实施重大军民融合项目23个，新增军工资质企业8家。供给侧结构性改革深入推进，钢铁落后产能全部退出，新增直购电、直供气企业372户，降低企业生产成本近4亿元。重点领域改革扎实推进，国资国企整合重组改革深入实施，“三供一业”分离移交改革走在全省前列，全面完成农村土地确权颁证，旌阳、什邡入选全国农村集体产权制度改革试点县，在全国首创闲置农房使用权流转“三书”模式。全域开放取得新进展。广泛开展友城交流合作，与苏州结为友好城市。三星堆古蜀文明展走进意大利、墨西哥，德阳杂技团《吴哥王朝》在柬埔寨正式开演。成功举办第八届中国月季展、第二届中国绿色产业博览会、第十五届中国医药博览会、中国全地形车锦标赛（罗江站）。140余家企业进入“一带一路”沿线国家和地区。与成都国际铁路港、广西防城港和钦州港签署战略合作协议。德阳国际铁路物流港一期建成投运，成功开行“蓉欧+”东盟国际测试班列，启动建设保税物流中心（B型）。

生态文明建设呈现新气象。狠抓中央和省环保督察反馈问题整改，全面办结中央环保督察“回头看”交办群众信访件186件。污染防治“十大攻坚战”扎实推进，空气质量优良率达78.6%、较基准年提高5.6个百分点，PM2.5浓度较基准年下降21.9%，整治“散乱污”企业1491家。土壤污染治理修复试点项目实施加快，完成磷石膏堆场整治20个、基本实现磷石膏“产消平衡”。落实河（湖）长制，实施重点流域治理项目230个、投资63.5亿元，国、省考核断面水质优良比例提高28.6个百分点。整治黑臭水体46公里，全面完成禁养区畜禽养殖场关闭（搬迁）工作，完成县级饮用水水源地保护区和68个行政村环境问题综合整治，罗江被列为全国农村人居环境试点县。环保基础设施不断完善，全面启动城乡生活污水处理厂建设、建成投运38个，柳沙堰、绵远河等城市污水处理厂提标扩能加快，生活垃圾焚烧发电项目竣工试运行，城市生活垃圾无害化处置率达100%。新增森林面积9702亩、森林覆盖率达24.8%，大熊猫国家公园生态复绿5.3万平方米。节能减排财政政策综合示范城市通过国家验收，累计获国省奖励15.9亿元、为建市以来最大单项财政奖励。

民生福祉获得新改善。全力推进脱贫攻坚。投入财政专项扶贫资金2.13亿元，改造建档立卡贫困户危房2681户、土坯房5023户，“五改三建”3.27万户，全市减贫1.31万人。成立省扶贫基金会德阳分会，募集款物2.32亿元。设立贫困村产业扶持基金，支持中江307个市级贫困村集体经济发展。对口帮扶藏区彝区工作成效明显，实施年度项目197个、投入财政资金7446.8万元，派驻干部人才848人。全面完成十项民生工程和20件民生实事。城镇新增就业4.53万人，城镇登记失业率3.71%。各类社会保险参保率达95%，城乡居民医疗保险政策范围内住院费报销比例达76.6%。城镇职工养老待遇实现“十四连增”，建立70周岁以上老人意外伤害保险制度。启动市儿童福利院建设。改造农村危房5079户、农村土坯房3万户、城镇棚户区7853套。

社会事业得到新发展。蝉联国家卫生城市，启动新一轮全国文明城市创建。教育事业加快发展。开工建设幼儿园9所、小学5所，改善农村义务教育薄弱学校基本办学条件28所，消除大班额93个，启动高中阶段学校考试招生制度改革，学前教育科研成果获国家二等奖。西南财大天府学院罗江校区、德阳天立学校、德阳城市轨道交通职业学院、德阳科贸职业学院建成招生。医药卫生体制改革纵深推进，县域内就诊率居全省首位，电子健康卡市（州）首发，“健康德阳APP”正式上线，罗江医共体模式成为全省经验，什邡、绵竹率先达到血吸虫病消除标准，旌阳被评为全国中医药先进县。文化事业繁荣发展，“三馆一站”全部免费开放，成功举办第四届中国青年志愿服务项目大赛暨志愿服务交流会、“一带一路”川菜川剧国际文化周、“百馆联动”等活动，成功创建全省首批“高清四川·智慧广电”示范市，《向阳公社摘牌记》获国家纪录片最佳作品奖。全民健身活动蓬勃开展，第13届省运会获金牌19枚，绵竹获全国群众体育活动先进县，新建农村体育中心户30个、全民健身苑14片。社会治理扎实推进，有效防控化解金融、政府债务、信访稳定、食品药品、非洲猪瘟疫情等重点领域风险，职业健康示范园区建设项目获全省第一。落实安全生产责任制，事故起数下降21.8%、死亡人数下降18.7%，未发生较大以上生产安全事故。扫黑除恶专项斗争扎实推进，高水平承办省级抗震救灾综合演练，成功应对“7·11”特大暴雨洪灾。大力支持国防和军队建设，双拥共建巩固发展，“德阳舰”正式入列。

政府自身建设展现新作为。依法行政扎实推进，认真执行人大及其常委会决议决定，自觉接受政协民主监督，办理人大代表建议178件、政协提案281件，邀请人大代表、政协委员36人次旁听市政府常务会，首部地方性实体法规《德阳市城市管理条例》开始实施，配合完成《德阳市物业管理条例》立法，提请审议《德阳市绵竹年画保护和发展条例》，制定首部政府规章《德阳市生活垃圾分类管理办法》，“法律七进”深入开展，办理行政复议91件。深化“放管服”改革，在全省率先实现“三十七证合一”，“最多跑一次”事项率达93.6%，企业开办审批时间压缩至3个工作日，重点投资建设工程项目审批压缩至92个工作日。建立重点工作月度调度会制度。积极回应群众关切，办理市长信箱3168件、办理率达100%。严格落实全面从严治党新要求，深入落实党风廉政建设“一岗双责”，坚决执行中央八项规定精神、省市十项规定及其实施细则，开播《德阳政事廉连问》，营造良好政治生态和干事创业氛围。

统计调查、审计、档案、民族宗教、住房公积金、人防、保密、地方志、气象、老龄、妇女儿童、青少年、残疾人、对台、哲学社会科学、红十字会等工作取得新成绩。

各位代表！成绩来之不易，这是习近平新时代中国特色社会主义思想正确指引的结果，是党中央国务院、省委省政府和市委坚强领导、科学决策的结果，是全市上下团结奋斗、顽强拼搏的结果。在此，我代表市人民政府，向市人大代表、政协委员、各民主党派、工商联、无党派人士、各人民团体、离退休老同志和各界朋友，向驻德解放军、武警官兵、政法干警，向在各个岗位上辛勤工作的全市人民和在德投资者、创业者、建设者，表示衷心的感谢，并致以崇高的敬意！

在看到成绩的同时，我们也清醒认识到，全市经济社会发展仍然存在不少问题和薄弱环节：发展质量不高，现代产业体系有待加快构建，传统产业转型升级任务较重，战略性新兴产业培育不够充分，现代服务业发展还有很大空间。发展动力不足，县域经济综合竞争力亟待增强，城市经济需加快发展，民营经济有待做强做大，重大产业项目引育还需持续突破。发展环境不优，资源环境约束趋紧，污染防治任务艰巨，营商环境有待提升，干部开拓创新、抢抓机遇、狠抓落实的能力有待进一步增强。民生领域还有不少短板，文化、教育、医疗、社保等公共服务与群众期盼仍有差距等等。对此，我们将高度重视，坚持问题导向、目标导向、实效导向，以逆水行舟、爬坡过坎的决心攻坚破难，以勇于担当、敢于负责的精神实干苦干，以务实创新、管用见效的举措加快发展，用干部的奋斗指数换取德阳高质量发展指数，用工作的辛苦指数增添群众的幸福指数！

2019年工作安排

2019年是建国70周年，是全面建成小康社会关键之年，也是倾力打拼全省经济副中心城市的强基之年。省委十一届三次全会将德阳确立为区域中心城市，赋予“打造世界级重大装备制造基地”“推动成德同城化突破”等十大战略任务，市委八届七次全会为德阳高质量发展描绘了宏伟蓝图，我们要把思想行动统一到省委市委决策部署上来，咬定目标、坚定信心，砥砺奋进、狠抓落实，在全省高质量发展中保持领先、实现跨越。

今年政府工作总体要求是：以习近平新时代中国特色社会主义思想为指导，认真贯彻党的十九大和十九届二中、三中全会以及中央经济工作会精神，全面落实省委十一届三次、四次全会和市委八届七次、八次全会部署要求，统筹推进“五位一体”总体布局，协调推进“四个全面”战略布局，坚持稳中求进工作总基调，贯彻新发展理念，落实高质量发展要求，以供给侧结构性改革为主线，围绕打造“四张名片”，全面实施“七大行动”，着力打好三大攻坚战，持续深化市场化改革、扩大高水平开放，统筹推进稳增长、促改革、调结构、惠民生、防风险各项工作，促进经济社会持续健康发展，加快建设全省经济副中心城市。

今年经济社会发展主要预期目标是：地区生产总值增长8.5%，全社会固定资产投资增长12%，一般公共预算收入同口径增长8.7%，规上工业增加值增长10%，服务业增加值增长10%，社会消费品零售总额增长11.5%，城乡居民人均可支配收入分别增长8.5%和9%，环境保护和节能减排达到国家和省的要求。

围绕上述目标，重点抓好以下九方面工作：

一、着力抓好创新引领，促进经济高质量发展

充分发挥实体经济对稳增长的支撑作用，坚持先进制造业和现代服务业双轮驱动、深度融合，确保全市经济高质量发展稳中有进、稳中向好。

加快建设先进制造业强市。工业要继续扛大旗、挑大梁。围绕打造世界级重大装备制造基地，加快构建“5+5+1”现代产业体系。提质振兴“五大主导产业”，加快设备换芯、生产换线、产品换代，着力延链、强链、补链，做强做大产业集群，装备制造业产值突破1400亿元、增长13%。培育壮大“五大新兴产业”，支持中国民航飞行学院建设世界一流特色大学，加快建设什邡军民融合通用航空机场，着力引进重大项目，力争通用航空产业实现突破性进展；打造石墨烯、铝空动力、中国锂都（德阿产业园）等专业园区，不断壮大新材料、新能源、轨道交通、节能环保产业，战略性新兴产业总产值突破1000亿元、占工业比重提高2个百分点。大力发展数字经济，推动新一代互联网等信息基础设施建设，培育大数据、云计算、物联网、工业互联网、人工智能等新兴产业，电子信息产业增长25%，建成智能制造试点示范项目6个。全面推进园区改革创新发展，落实“六个统筹”,加快德阳经开区东区（成德工业园）、德阳高新区北区（罗江园区）规划建设，奋力打造德阳经开区、德阳高新区“双千亿”园区，建设“五百亿”园区6个，新增省级高新区2家。大力培育骨干企业，培育引进“双百企业”2户，新增上市企业1户、行业单项冠军企业1户、瞪羚企业3户，净增规上工业企业50户。狠抓工业项目，实施重点工业项目181个、完成投资85亿元，开工建设深兰科技西南总部等53个项目，加快建设聚亿重工透平压缩机生产线等42个项目，竣工投产华磷科技磷石膏综合利用等60个项目，促进亿达产业园等一批重大项目落地。

加快建设现代服务业强市。大力发展现代服务业，着力构建现代服务业新体系，打造区域总部、商贸、物流、金融中心，服务业占GDP比重提高2个百分点。深入实施服务业“三百工程”，编制现代服务业集聚区规划，建设15个服务业集聚区、6个智慧商圈、10条特色街区，加快京东西南智能运营结算中心、北城世贸城等30个重大项目建设，新增限上商贸企业和规上服务业企业100家。提质发展生产性服务业，争创省级服务业和制造业双轮驱动发展试点，重点发展现代物流、电子商务、科技服务等产业。创新发展生活性服务业，深化与成都在医疗健康、教育培训等领域合作，大力发展分享经济、数字创意、智能家居等新业态新模式。加快发展现代金融业，扩大直接融资规模和比重，人民币存贷款余额增长9%、存贷比提高2个百分点，新引进金融机构3家。鼓励支持建筑企业向房地产、设计、营销等上下游产业链拓展，实现建筑业产值474亿元、增长15%。挖掘消费潜力，实施消费提质计划，改善消费环境，创建“放心舒心消费城市”，扩大绿色消费、品质消费、信息消费、假日消费，办好惠民购物全川行等节会活动。

全力争创国家创新型城市。新增国家高新技术企业10家，高新技术产业主营业务收入增长12%以上，R&D占GDP比重达到2.8%。提升企业自主创新能力，发挥国机重装、东电、东汽、宏华等企业在转型升级、创新引领中的带动作用，推动大中小企业联合创新、协同发展，完成国家50MW重型燃机等一批重点科技攻关。完善产学研协同创新体系，加快建设成德绵国家科技成果转移转化示范区，打造德阳（西门子）智能制造创新中心等10个创新平台，促进装备制造业等5个技术创新联盟发展，新增重大科技成果转化项目30个以上。实施“双创”升级行动，大力开展“德创英才引育计划”“德阳工匠培育计划”，引进培育高层次人才100人、专业孵化机构10个、创新创业团队10个。深化国家知识产权示范城市建设，抓好科技与金融结合试点，全面推行专利商标质押融资，建立重装产业知识产权运营服务平台、专利培育示范中心，新增国家授权发明专利420件。

打好防范化解重大风险攻坚战。牢固树立防风险底线思维，坚决守住不发生系统性、区域性风险的底线。打好金融风险防控攻坚战，加强金融生态环境建设，严厉打击非法集资、金融诈骗等违法活动，不良贷款率控制在警戒线5%以内。防范化解政府债务风险，有序化解存量债务，严禁违法违规举债、担保等行为，隐性债务“只减不增”，确保政府债务总体可控。防范化解企业投资债务风险，支持市场化法治化债转股和兼并重组，引导企业有效规避投资风险。防范化解房地产领域风险，完善住房市场体系和保障体系，促进房地产业平稳健康发展。

做好稳增长工作、促进全市经济高质量发展，要在“巩固、增强、提升、畅通”上下功夫，通盘考虑、突出重点、抓住关键。要加强全市统筹，建立重大项目“资金池”“土地池”，全力推动重大项目“月调度、季拉练”，强化环境容量、水、能源、物流等要素保障，加强项目储备和建设，精心谋划一批补短板项目，严格考核项目投资完成率和工程进度。

二、着力抓好营商环境，促进民营经济健康发展

坚定落实“两个毫不动摇、三个没有变”，着力破解“三门三山”，切实做到政策落实到位、减税降费兑现到位、帮扶企业服务到位，为民营经济发展营造更好环境。

支持民营企业发展壮大。全面落实国省市民营经济发展政策，支持引导民营企业做强做大，民营经济增加值达1300亿元、占GDP比重提高到57%，新增省级百强民营企业2户、民营经济市场主体1万户。实行本土民营企业新增投资享受招商引资同等政策，大力支持产业联盟、行业商（协）会发展。促进中小民营企业向“专精特新”发展，支持领军型民营企业跨行业、跨地区、跨所有制发展，鼓励企业上市融资、发行债券、兼并重组。大力开展“春风行动”，切实解决一批民营企业难点、堵点、痛点问题。关心民营企业家成长，实施民营企业家培育计划、德商返乡兴业计划，建立民营企业家旁听市政府常务会、季度恳谈会制度，真正把民营企业家当“自己人”。

降低民营企业经营成本。切实减费降税。进一步减少政府涉企行政事业性收费和政府定价的经营服务性收费，落实民营企业社会保险阶段性降费率、失业保险稳岗补贴等政策。降低要素成本。实施先租后让弹性供地政策，保障好民营企业重点投资项目用地需求。全面落实工业企业用能普惠政策，扩大直供气用户范围，为规上民营企业配备专属用电秘书，新增直购电企业43户。降低进出口环节成本，加大东盟国际班列、蓉欧快铁支持力度，推行国际贸易“单一窗口”应用。降低融资成本。健全政银企融资对接机制，启动建设综合金融服务平台，用好再贷款、再贴现、降准资金和应急转贷资金。扩大应收账款质押融资、“园保贷”“助保贷”规模，打造装备制造、医药等供应链金融，引导金融机构加大对民营企业贷款投放，防止盲目抽贷断贷，民营企业贷款增速达15%以上。完善企业全生命周期创业投资体系，建立民营企业发展基金和纾困基金。

开展营商环境提升年活动。以增强微观主体活力为重点，建立公平开放透明的市场规则和法治化营商环境。建立民营企业参与营商环境政策制定和评价机制，完善民营企业问题快速处置响应机制。全面实施市场准入负面清单制度，破除在产权保护、要素配置等方面歧视性限制和隐性障碍。持续深化“放管服”改革，推行审批服务“无差别全科受理”“最多跑一次”“全程网办”，优化全程代办机制，进一步理顺市区两级审批权限，推行企业办事“不出园区”，实现“当天开业、百天开工”。完善综合监管机制，建立监管对象追溯体系，推进“双随机、一公开”。加强行业自律，加快全社会信用体系建设。构建亲清新型政商关系，营造民营经济健康发展的法治环境、市场环境、社会环境。

三、着力抓好协调发展，构建区域发展新格局

落实“一干多支”发展战略，加快成德同城化、德绵一体化，强化市级统筹、市县联动，进一步增强县域经济综合实力。

推进成德同城化率先突破。细化落实成德眉资同城化合作协议，实施好75项重点合作项目和重点工作。强化规划编制协同，共同编制“成德同城化五年发展规划”“成都大都市圈规划”和专项规划，构建“1+N”规划体系。打造环成都经济圈通勤最佳城市，实施交通加密成网计划，加快构建“7高8快9轨”交通体系。提前建成成都经济区环线德简段，开工建设成德绵高速扩容线、成南高速扩容线、天府国际机场快速通道等重大项目，加快建设成都经济区环线德都段，规划建设德阳经开区至成德工业园的工业大道。重点推进天府大道北延线建设，完成投资30%，同步编制“天府大道北延线城市总体规划”，打造“百里中轴”经济带。编制“城市综合轨道交通体系规划”，力争开工建设对接成都的城际轨道新线，推动建设成彭什绵轨道交通。建成投运德阳城市候机楼。深化成德边界产业合作，推动成德工业园与淮州新城协同发展，促进成德产业集群成链发展，共同打造龙泉山现代产业走廊、环龙门山旅游示范带。与成都一体打造“三星堆·金沙”世界级文旅品牌，实现平台共享、联合运营。健全成德两地市场协同机制，推进企业、产品市场准入互信互认。抢抓成都北改、中优机遇，承接专业市场、批发市场、物流园区转移，融入成都国际消费城市建设。加快公共服务共享，开展成德大数据联盟合作，持续推进成德公交、医保、社保“一卡通”。

加快德绵一体化进程。落实德绵一体化发展合作协议。加快基础设施建设，开工建设德绵遂高速公路，积极推进成德绵大道、德罗干道北延线、德阳到绵阳机场快通等项目，推动德绵动车公交化，开通罗江金山至绵阳高新区跨市公交。深化产业合作，支持德绵边界产业合作，推动金山军民融合产业园融入绵阳军民融合创新驱动核心示范区。建立协同创新联席会议制度，推动教育、卫生、医疗等一体化合作，放宽两地公积金异地购房使用政策，探索“不迁户口、不转关系、来去自由”人才共享政策。

提升中心城市功能品质。坚持精准规划。加快编制国土空间规划和各类专项规划，力争用两年时间实现多规合一、全域覆盖。规划设计文化、商务、行政、市民中心并适时启动建设，提高中心城市“首位度”和辐射带动力。大力发展城市经济，优化城市功能布局，拓展城市发展空间，加快“两区一谷一城”建设，重点建设北部“旌北新区”，着力打造以高铁经济、现代金融、高端商务、文化创意为主的高铁门户新城；优化提升南部“旌南新区”，着力打造以总部经济、科技研发、商务会展、高端制造为主的产城融合新区；加快建设东部“天府生态智谷”，着力打造以国际教育、医疗康养、文化旅游、智慧产业为主的高端产业生态新区；高标准规划建设西部“天府旌城”，着力打造以天府大道北延线为城市中轴、以石亭江人工湿地为生态本底、以现代产业为支撑的公园城市新区。支持罗江区加快发展，着力打造德阳北部新城。坚持精致建设。争创全省公园城市试点，实施城市“引水、增绿、置景”计划，开工建设锦绣天府公园、高铁景观中轴带、青衣江桥以北旌湖两岸景观带，建成柳梢堰湿地公园和8个“邮票公园”。实施旧城提质增能计划，改造北光、文庙等老旧小区和一批老旧市场，开工建设渭河路农贸市场、餐厨垃圾处理厂，新（改）建公共厕所381个；抓好海绵城市试点，提升城市防洪能力；启动二重、工农村片区等老工业区城市记忆项目规划建设。坚持精细管理。全面施行《德阳市城市管理条例》《德阳市物业管理条例》，推进生活垃圾分类全国示范市建设。优化城市综合交通体系，实施缓堵保畅工程，规划建设德阳绕城高速公路环线，开工建设青衣江东路延长线、金沙江路提升改造等项目，开通南北大站公交、实现半小时通达，综合治理泰山路、岷江桥等堵点，打通断头路8条，增设城市主干道“安全岛”。推进城乡公交一体化。加强智慧交通、智慧城管等智慧城市建设。全面开展城市推广，提升城市知晓度、美誉度。

提高县域经济综合竞争力。牢固树立全市“一盘棋”思想，进一步厘清市、县（市、区）、乡镇（街道）权责划分，统筹推进县域经济竞合发展、错位发展、特色发展。编制全域德阳城乡空间体系功能区规划、区域发展专项规划。实施县域经济提质升位行动，推进“小县优城”提升计划，制定县域经济工作方案和考核激励办法，鼓励争创全省县域经济发展强县、先进县和进步县。坚持“一县一园、一主两辅”产业布局，加大重大产业项目引进，实现具有龙头带动作用的特别重大项目新突破，力求“一项兴一业、一业定乾坤”。开工建设德什、德绵等干道，加快建设德罗、德中等干道，构建“三环多轴”市域一体化交通体系，打造市域“半小时”通勤圈。积极推进广汉撤市设区。扎实开展“百镇建设行动”，新增省级以上特色小城镇1个。

四、着力抓好开放拓展，打造内陆改革开放高地

改革开放是发展的动力之本、活力之源。实现经济繁荣、人民富裕，必须用好改革开放这个“法宝”，向改革要“红利”、向开放借“东风”。

狠抓重大改革突破。打好全面深化改革攻坚战，持续深化行政体制、投融资体制、商事制度、社会治理、民生保障等重点领域改革，着力破解高质量发展中的瓶颈制约因素，全面完成280项改革任务。巩固提升全面创新改革成果，持续加强创新平台搭建、重大科技攻关、创新人才支撑、科技成果转化，不断深化军转民、民参军领域重点改革，加快建设“三园四基地”军民融合产业园，创建国家军民融合创新示范区，军民融合产业产值突破900亿元。深化国资国企改革，全面完成“三供一业”分离移交，持续推进市属国有企业“人资分离”改革，加快发展混合所有制经济，支持市产投、市建投做强做大。制定退出办法，加快处置“僵尸企业”。全面实施预算绩效管理，继续推动市县财政改革两年攻坚计划。

打造内陆开放门户。深入实施“四向拓展、全域开放”，积极融入“一带一路”建设和长江经济带发展，推动更多德阳品牌、产品、技术“走出去”。实施“自贸进园区专项行动”，建设中国（四川）自贸试验区协同改革先行区，创建国家开放口岸。推进综合保税区、保税物流中心（B型）建设。加快建设海峡两岸产业合作区德阳产业园。强化南向开放大通道建设，探索与钦州港、防城港共建内陆“无水港”。办好首届中国装备智造国际博览会、第二届四川国际航空航天展览会等重大活动，积极参加中国国际进口博览会、“万企出国门”“千企行丝路”等系列活动。

突出抓好招商引资招才引智。实施产业项目招引攻坚行动，瞄准京津冀、长三角、粤港澳大湾区等重点区域，加大龙头企业、龙头项目、核心团队的招引力度，力争高端装备、通用航空、新一代信息技术、现代物流、新材料、电子商务等重点产业取得新突破，引进100亿元以上特别重大项目、30亿元以上重大项目14个以上，到位市外资金770亿元。完善“市统筹、县落地”招商工作机制，增加考核权重，强化激励约束机制，实行“月通报、季拉练”，设立投资促进服务中心，建立专业化招商队伍和市县两级信息共享平台，构建“130”立体招商网络。积极参加中外知名企业四川行、西部国际投资大会、科博会等活动。

五、着力抓好乡村振兴，建设西部现代农业先行示范市

高标准编制全市乡村振兴战略规划，统筹实施好乡村“五个振兴”，争当全省乡村振兴排头兵。

坚决打好脱贫攻坚战。始终把脱贫攻坚作为最大的政治责任、最大的民生工程、最大的发展机遇，聚焦“两不愁、三保障”，聚焦5200户9709人脱贫目标，大力实施中江县脱贫攻坚，其他区（市）全面脱贫。着力产业扶贫，用好贫困村产业扶持基金，推广股权合作、订单帮扶、生产托管、土地入股等模式，新建农业产业园区20个。着力就业扶贫，确保有劳动能力和就业意愿的贫困家庭至少1人实现就业。着力教育扶贫，抓好“控辍保学”责任和教育资助政策落实，实现无一个贫困学生因贫辍学。着力医疗扶贫，深入开展健康扶贫“五大行动”，落实“十免四补助”，确保贫困人口县域内住院个人支付比例控制在10%以内。着力社保扶贫，建立以社会保险、社会救助、社会福利制度为主体，以慈善帮扶为辅助的保障体系。着力住房改善，继续实施贫困户“五改三建”，易地扶贫搬迁9270人。着力饮水安全，解决2000户建档立卡贫困户饮水安全问题。着力“回头看”“回头帮”，做好已脱贫退出的贫困人口、市级贫困村的巩固提升工作。抓好对口帮扶藏区彝区工作，实施年度项目188个，落实财政帮扶资金7970万元。

推动农业高质量发展。规划建设350公里外环现代农业产业带和150公里内环都市休闲农业产业带，各县（市、区）打造具有全省影响力的乡村振兴精品项目1-2个。构建“2531”现代农业产业体系，探索“特色小镇+农业园区+专合社”模式，争创国家级现代农业产业园区1个、省级2-3个。强化农业科技支撑，组建市农科院，建立农业院士工作站1-2个，依托乡村振兴农民大学培养新型职业农民2000人，开展农业大师评比活动。实施品牌兴农战略，做优“蜀道”品牌，新增“三品一标”农产品20个，农业企业自主品牌50个。大力推进农产品精深加工，提升农产品附加值。加强农产品质量安全监管，增加优质绿色农产品供给，落实粮食安全行政首长负责制，筑牢非洲猪瘟防控屏障，实施农业综合执法改革，净化农资市场环境，建设园区农产品质量检测平台，确保全市农产品抽检合格率达98%以上。培育壮大农业新型经营主体，规范发展农民专业合作社100家、家庭农场50家。

切实改善农村人居环境。实施农村人居环境整治三年行动，开展“千村示范、万村整治”工程，建设一批“美丽四川·宜居乡村”达标村和“四川最美古村落”，争创省级乡村振兴先进县1个、乡镇2个以上、示范村15个以上，推进有条件的乡镇、行政村撤并整合。建立健全村镇规划建设管理机构，建立乡村规划师制度，实施村容村貌提升“六化”工程，抓好农村垃圾、污水、厕所“三大革命”和彩钢棚整治，生活污水、垃圾治理率分别达50%、95%，卫生厕所普及率达80%，新（改）建农村公路400公里，打通一批农村断头路。打造精品亮点，启动建设成德同城化乡村振兴先行示范区，加快建设成德乡村振兴示范走廊。

加快推进农业农村改革。大力推行农村“三变”改革，加强农村“三资”管理，全面开展农村集体资产股份合作制改革，完成村集体经济组织登记颁证90%以上。推进农村承包地、宅基地“三权分置”改革，大力推广闲置农房使用权流转“三书”模式，增加农民财产性收入。依托成都农交所德阳所，建立健全市县乡村四级农村产权流转交易市场体系、市县两级农村担保体系和农资产品交易中心。强化乡村振兴考评激励和投入保障，设立乡村振兴发展基金，组建市农投公司，创建农村抵贷收储联盟，创新经济林木、集体资产抵押融资模式。

六、着力抓好文化提升，全力打造古蜀文化名城

深度挖掘、传承和利用古蜀文化资源，弘扬文化精髓、塑造城市精神、开发文化精品，切实推动文化繁荣兴盛。

打造古蜀文化产业带。做优做强古蜀文化、三国文化、德孝文化等品牌，高标准建设三星堆文化产业园，打造绵竹“年文化”产业园、白马关三国文化产业园等省级文化园区。实施三星堆古蜀文明传承创新工程，与金沙遗址联合申报世界文化遗产，协同打造“三星堆-金沙-大熊猫基地-九寨沟”精品旅游线。支持三星堆遗址创建国家5A级景区，办好三星堆戏剧季、面具节、大祭祀等活动，打造世界级古蜀文化IP。

大力发展文旅产业。促进“文化+”“旅游+”融合发展，加快构建“一城两带、一环三廊”全域旅游发展格局。举办全市文化和旅游发展大会，实施文化街区、文化园区、特色小镇等重点工程，开工建设雪茄小镇、冰雪小镇、中国挂面第一村等项目。支持绵竹创建国家全域旅游示范区、各地争创天府旅游名县。大力发展乡村旅游，积极培育精品民宿、田园综合体、森林康养、旅游小镇等新产业新业态。优化提升旅游环境，加大违法违规行为整治力度。实现旅游总收入460亿元、增长20%。

提升公共文化服务水平。培育和践行社会主义核心价值观，加强新时代精神文明建设，以全国文明城市创建为引领，围绕“提升突破，争先晋位”，实施创城“五大工程”，加快构建崇德向善的文明环境，群众满意度测评达90%以上。加快科技馆、规划馆、会展中心、大数据“智慧之心”等的规划建设，支持什邡、绵竹创建省级公共文化服务示范县。加强对古蜀文化、民俗文化、“三线建设”文化等的提炼、转化，挖掘脱贫攻坚、高质量发展等时代题材，创作更多精品力作。推进公共文化服务标准化建设，健全市县乡村四级公共文化服务网络。

七、着力抓好生态优先，增强可持续发展承载力

严守生态保护红线、环境质量底线、资源利用上线，坚决打好污染防治“十大攻坚战”，建设人与自然和谐共生的美丽幸福家园，让德阳山水“颜值”更高、大地“气质”更佳。

精准发力整治环境突出问题。结合中央环保督察“回头看”反馈问题，以钉钉子精神全面抓好问题整改，完成28项中央反馈问题整改、213项省督问题整改。实行最严格的环保制度，完成全国第二次污染源普查工作。实施大气污染防控“五大工程”，完成企业清洁生产审核22家，空气质量优良率达76.9%，PM2.5浓度较基准年下降14.8%。建设土壤风险管控区和先行示范区，巩固提升磷石膏治理成果，狠抓新市工业园等重点区域环境问题综合整治，全面完成农业用地土壤环境质量状况调查。深化“环保+公检法”执法模式，建立企业环境信用评价和环境污染“红黑名单”制度。增强服务意识，帮助企业制定环境治理解决方案。

聚焦聚力治理流域水环境。深入落实河（湖）长制，大力推进沱江生态廊道建设，国、省考核断面水质优良比例达到71.4%。积极申报城市黑臭水体治理示范市，完成中心城区穿城堰等4条黑臭水体整治任务，建立黑臭水体防治长效机制。加快建设华强沟水库、旌东水厂，开工建设西郊水厂4万吨扩能、周家坝湿地公园、广汉三星湖，做好高景关水库和绵远河上游梯级蓄水保水工程前期工作。开展乡镇饮用水水源地和农家乐环境问题排查整治。加强污水处理设施建设，完成柳沙堰、绵远河污水处理厂提标扩能，加快石亭江、天元污水处理厂提标扩能，推进城乡污水处理设施BOT项目，市区、县城和建制镇污水处理率分别达95%、85%、50%。建立健全污染防治联防联控机制，完善跨流域、跨区域环境应急联动机制。

持续用力推进绿色低碳循环发展。实施大规模绿化行动，做好国家森林城市复查迎检工作，抓好天然林保护二期建设、大熊猫国家公园体制试点，森林覆盖率达24.9%。巩固节能减排示范市成果，健全排污权、碳排放权等市场化交易机制。落实国家重点用能单位“百千万”行动，推进国家级园区循环化改造示范试点，争创国家资源循环化利用示范基地、国家生态工业示范园区。建设中江沼源循环农业产业园。推动餐厨垃圾和污泥无害化处置，建设并推广生物质垃圾焚烧发电一体化示范项目，提升危险废物、固体废物、医疗废物处置能力。

八、着力抓好民生提质，增强群众获得感幸福感

民生是为政之本，我们将念之再三、铭之肺腑，兜底线、保基本、优品质，既量力而行、又尽力而为，不断满足人民群众对美好生活的向往。

切实提高社会保障水平。实施好30件民生实事，民生支出占一般公共预算支出65%以上。把稳就业摆在突出位置，抓好高校毕业生、农民工、退役军人等重点群体就业创业，城镇新增就业4.5万人，城镇登记失业率控制在4%以内。实施全民参保计划，推行城镇职工大病保险制度，各项社会保险综合参保率达96%。落实社会救助、社会福利等制度，关心关爱留守儿童，加快建设市儿童福利院和精神病人社会福利院，推进养老服务体系建设，提升“日间照料中心”服务能力和水平，深化“量体裁衣”式残疾人服务，建设“德善之城”。加强农民工服务保障，做好维护权益、搭建平台等工作，春节期间全覆盖走访返乡农民工，把政策和服务送上门。改造农村危房5786户、农村土坯房3.76万户、城镇棚户区6464套。

全面推进社会事业发展。优先发展教育事业，实施“名师名校”工程，新（续）建幼儿园12所（其中中心城区公办5所）、中小学7所，改善农村义务教育薄弱学校基本办学条件16所，消除大班额55个，打造名优普通高中3-5所，做好高考综合改革工作；推动职业教育国际化，支持职业院校争创国省示范特色院校及专业，加快建设成都外国语学校（北区）、德阳巴川公学、电子科大成都学院什邡校区。深化“健康德阳”建设，实施“名医名院”工程，推进国家级医养结合和安宁疗护试点，促进并规范社会办医，加快市医院城北院区、市妇女儿童专科医院等项目建设，推动6家医疗机构达三级医院标准。积极申办第15届省运会和重大体育赛事，打造群众体育“六个身边”工程。加强国防动员、国防后备力量建设。做好第三次全国国土调查和第四次全国经济普查。

大力提升社会治理现代化水平。争创全国综治“长安杯”，建设更高水平的“平安德阳”。深入实施“主动警务战略”，创新“公调对接”“访调对接”矛盾纠纷调处新机制，推进综治中心、雪亮工程建设。深入开展扫黑除恶、禁毒防艾专项斗争，实施“两打三控”专项行动。坚持和发扬新时代“枫桥经验”，落实“四级三三制”调解制度，加强重点人群服务管理。依法管理民族宗教事务。探索创新基层社会治理方式途径，推进基层社会治理现代化。扎实抓好食品药品安全，加强抽检监测和执法力度。深入推进应急管理机构改革，提高防灾减灾救灾和安全生产保障能力，狠抓重点行业领域专项整治，坚决遏制重特大事故发生，构建新时代应急救援体系。

各位代表，办好民生事，须先听民声。从今年起，每年聚焦群众“急难愁盼”民生难题，通过电视、报刊、网络等媒体，面向社会公开征集“十大民生焦点实事”，公开实施进度，公开办理结果，扎扎实实办上几件群众最关心最急切的实事，让发展实绩更有温度，民生答卷更有厚度！

九、着力抓好政府建设，增强政府现代化治理能力

打铁必须自身硬。牢固树立“四个意识”，坚决践行“两个维护”，全面落实市委《关于进一步解放思想改进作风狠抓落实的决定》，全面提升政府服务能力和水平。

着力建设法治政府。大力弘扬宪法精神，加强依法行政，把政府各项工作全面纳入法治轨道。认真执行市人大及其常委会决议决定，自觉接受市人大及其常委会法律监督、工作监督和市政协的民主监督，办好人大代表建议、政协委员提案。主动接受民主党派、人民群众和社会舆论监督。完善权力清单、责任清单和负面清单。做好地方性法规起草和政府规章制定。推进公共法律服务体系建设，深化“七五”普法。高质量完成机构改革任务。

着力建设创新政府。弘扬“敢为人先”精神，破除固步自封、墨守成规观念，鼓励创造性贯彻落实，激励干部勇于攻坚破难题、敢于担当促发展、善作善为谋实绩。发挥“头雁”效应，建立“五见”和“五个一”工作机制，扑下身子抓落实。推进新型智库建设，切实提高科学决策水平。积极运用新技术推动政府管理创新。完善正向激励机制，深化容错纠错防错、澄清保护、关心关爱机制,让改革创新者轻装上阵、实干争先。

着力建设效能政府。扎实开展“落实攻坚年”活动。力戒形式主义、官僚主义，重点治理表面文章、过度留痕、文山会海、督查检查过多过滥等现象，切实解决好表态多、调门高，行动少、落实差等突出问题。推进“互联网+政务服务”，市县两级政务服务事项网上可办率达到100%。强化行政绩效考核，综合运用信息化督查、第三方评估等方式，提高执行力和工作效能。

着力建设廉洁政府。坚定不移推动全面从严治党向纵深发展，深入落实中央八项规定精神、省市十项规定及其实施细则，持之以恒正风肃纪，营造风清气正的经济社会发展环境。深入推进政府系统党风廉政建设和反腐败工作，把权力关进制度的笼子，加强公共权力、公共资金、公共资源等的监管，强化重点领域审计和监督，管住关键事、管到关键处、管好关键人。

各位代表，新时代是奋斗者的时代，精彩德阳需你我齐心协力，魅力德阳靠大家同舟共济！让我们紧密团结在以习近平同志为核心的党中央周围，在省委、省政府和市委的坚强领导下，团结依靠全市人民，以永不懈怠的精神状态和一往无前的坚定决心，凝心聚力，抢抓机遇，将实干进行到底，将改革开放进行到底，奋力打拼全省经济副中心城市，以优异的成绩迎接新中国成立70周年！
